# Engineering biodegradable and multifunctional peptide-based polymers for gene delivery

**DOI:** 10.1186/1754-1611-7-25

**Published:** 2013-10-24

**Authors:** Julie Shi, Joan G Schellinger, Suzie H Pun

**Affiliations:** 1 Department of Bioengineering and Molecular Engineering & Sciences Institute, University of Washington, 3720 15th Ave NE, Seattle, WA 98195, USA

**Keywords:** Nonviral gene delivery, Peptide-polymers, Endosomal escape, Degradability, *N*-(2-hydroxypropyl)methacrylamide (HPMA)

## Abstract

The complex nature of *in vivo* gene transfer establishes the need for multifunctional delivery vectors capable of meeting these challenges. An additional consideration for clinical translation of synthetic delivery formulations is reproducibility and scale-up of materials. In this review, we summarize our work over the last five years in developing a modular approach for synthesizing peptide-based polymers. In these materials, bioactive peptides that address various barriers to gene delivery are copolymerized with a hydrophilic backbone of *N*-(2-hydroxypropyl)methacrylamide (HPMA) using reversible-addition fragmentation chain-transfer (RAFT) polymerization. We demonstrate that this synthetic approach results in well-defined, narrowly-disperse polymers with controllable composition and molecular weight. To date, we have investigated the effectiveness of various bioactive peptides for DNA condensation, endosomal escape, cell targeting, and degradability on gene transfer, as well as the impact of multivalency and polymer architecture on peptide bioactivity.

## 

Gene therapy has the potential to improve therapeutic outcomes for currently untreatable diseases. Viruses are naturally efficient vectors for gene therapy, but have encountered obstacles in clinical translation due to issues such as vector toxicity and immunogenicity, potential gene integration into oncogenic regions, and high production costs. Thus, nonviral materials, *e.g.* cationic lipids and polymers, have been extensively engineered as gene delivery vectors and are attractive alternatives to viral vectors because they tend to have improved safety profiles and lower costs of production [1]. However, nonviral vectors have not been as successful in attaining high transgene expression efficiencies *in vivo*. In order to enhance the gene transfer efficiency of these materials, several groups have explored the use of bioactive peptides to address various extracellular and intracellular barriers to nonviral gene delivery, such as cellular uptake, endosomal escape, cargo unpackaging, and nuclear translocation [2-5]. These multicomponent synthetic materials have been engineered to overcome these barriers for various applications [6], including delivery to neurons [7] and hepatocytes [8].

Recent advances in living polymerization techniques, such as reversible-addition fragmentation chain transfer (RAFT), have allowed the development of well-defined polymers with controlled architectures and quantitative monomer incorporation [9-11]. Peptide-polymer conjugates containing multiple peptides can be synthesized by grafting peptides to preformed polymers or by polymerization of peptide monomers. The grafting technique has been extensively reviewed elsewhere [12-14]. The Klok group demonstrated the synthesis of peptide brush copolymers by RAFT polymerization of coiled coil peptide motifs in 2010 [15].

In this review, we summarize our work in the development of copolymers consisting of *N*-(2-hydroxypropyl)methacrylamide (HPMA) and multiple pendant oligopeptides for gene delivery (Figure 1). Cationic peptide moieties were explored and optimized for DNA condensation. To improve biodegradability, environmentally-responsive linkers were incorporated into the HPMA copolymers. In addition, both buffering and membrane-disruptive peptides were evaluated for enhancing endosomal escape of the polyplexes. Throughout our work, we have also noted effects of polymer architecture and multivalency on transfection efficiency. Finally, we conclude with some therapeutic applications for this class of peptide-functionalized brush polymers that we have investigated.

**Figure 1:**
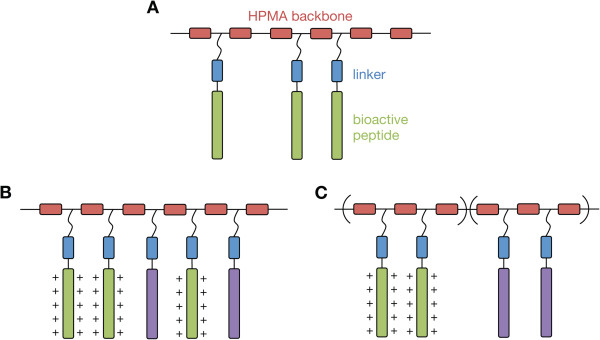
**Schematic of peptide-functionalized HPMA copolymers. (A)** Random-statistical copolymers of HPMA (red) and pendant bioactive peptides (green) with a linker (blue) between the hydrophilic backbone and the main peptide sequence. **(B)** Random-statistical copolymers of HPMA and multiple pendant peptides (cationic peptide in green; second bioactive peptide in purple). **(C)** Diblock copolymer of HPMA and multiple pendant peptides, where the cationic peptide is on one block, and a second bioactive peptide on a different block.

## Design of peptide-based polymers

### Nucleic acid condensation using basic peptides

Cationic vectors can be used to form electrostatic complexes with anionic nucleic acid, termed “polyplexes”, to protect the nucleic acid from serum and intracellular proteases. Early studies demonstrated the use of poly-l-lysine as a transfection agent to deliver plasmid DNA to achieve exogeneous protein expression [16]. Further work was conducted to optimize the oligolysine and polylysine residues for enhanced gene transfer, as well as introduce salt- and serum-stability into these carriers [17]. Since then, a number of cationic polymers have been developed for gene delivery applications [1,18]. In 1997, O’Brien-Simpson and coworkers reported a general method for the assembly of multi-peptide polymer constructs using acryloyl peptides by radical polymerization for vaccine development [19]. Thus, we hypothesized that this polymer architecture would enable a greater incorporation of peptide moieties and flexibility for multiple peptide incorporation for the development of gene delivery vectors.

**Figure 2:**
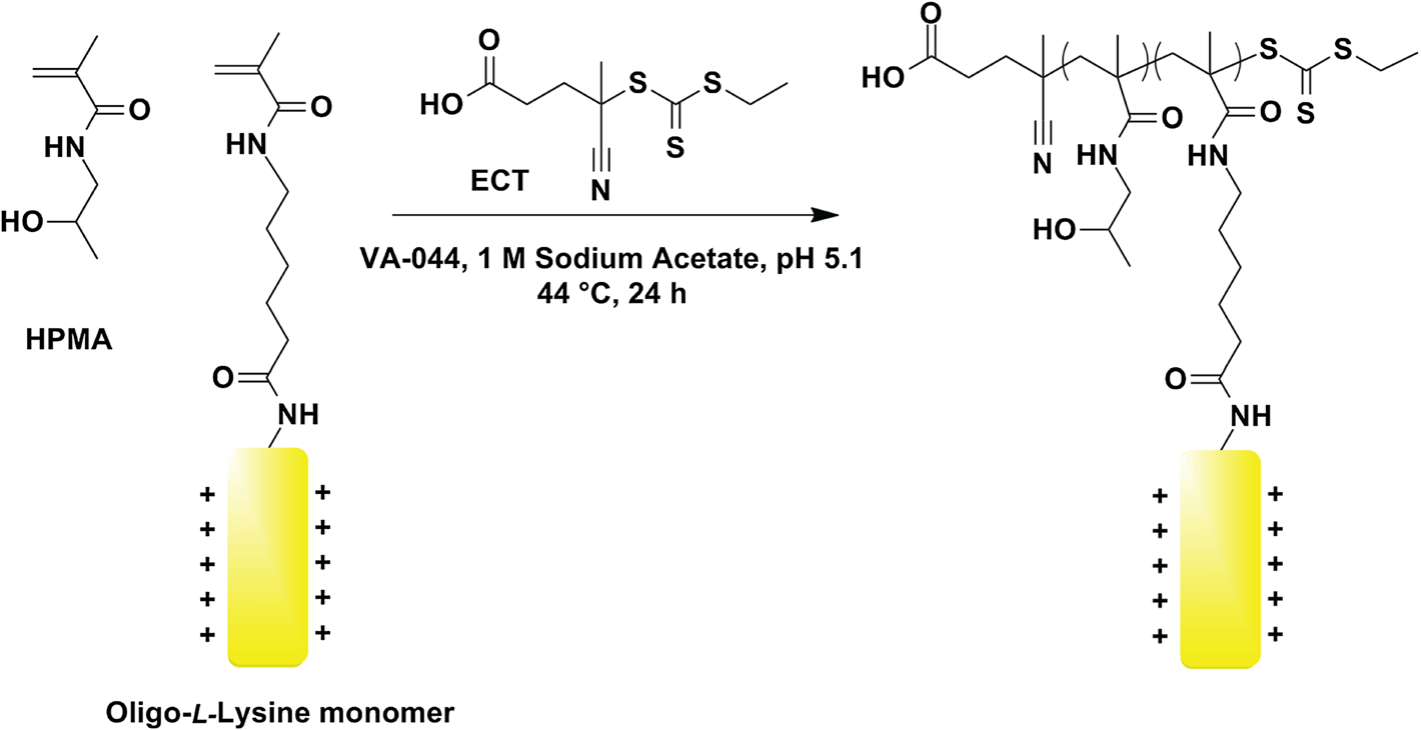
**Synthesis of statistical HPMA-oligolysine copolymers by RAFT polymerization.** HPMA and methacrylamido-functionalized oligolysine peptides are copolymerized under aqueous RAFT conditions using ethyl cyanovaleric trithiocarbonate (ECT) as the chain transfer agent (*CTA*) [71] and VA-044 as the initiator. The 6-carbon aminohexanoic acid (Ahx) is used as a linker between the pendant oligolysine peptide and the hydrophilic backbone.

As a starting point, HPMA was chosen to compose the hydrophilic backbone. HPMA has been widely used for the synthesis of polymer-drug conjugates due to its biocompatibility, as well as its synthetic versatility and flexibility since it has been used in a variety of polymerization techniques [20]. Therefore, we copolymerized short pendant oligolysine peptides with HPMA in a random-statistical brush polymer architecture using both free radical and RAFT polymerization approaches (Figure 2) [21,22]. Polymers synthesized by RAFT polymerization exhibited polydispersities closer to 1, better control over final polymer composition, and reduced toxicities [23]. We next optimized the peptide length and molecular weight of these statistical polymers generated by RAFT polymerization, and found that a peptide length of 10 lysines, a composition of 5 mmol lysine per gram polymer, and a molecular weight of ~60 kDa was optimal for high transfection efficiencies (comparable to that of branched polyethylenimine, or bPEI) and limited cytotoxicity [23]. Surprisingly, a 50% longer oligolysine peptide (K_15_) demonstrated poor transfection efficiencies despite similar lysine composition and molecular weight. Extensive studies on the mechanism of enhanced transfection of the optimized polymer (*p*[HPMA-*co*-K_10_]) over the poorly-performing polymer (*p*[HPMA-*co*-K_15_]) showed that polyplexes of larger aspect ratios (more rod-like) greatly reduced cellular uptake and subsequent transfection efficiency (Shi J, Choi JL, Chou B, Johnson RN, Schellinger JG, Pun SH: Effect of polyplex morphology on cellular uptake, intracellular trafficking, and transgene expression. Submitted). Previous studies have reported that polylysine length correlates to the aspect ratio of the lysine/DNA complexes, thus highlighting the importance of understanding the biophysical interaction of plasmid DNA with various cationic moieties. In order to reduce cytotoxicity, we explored the effect of reduced charge density by alternating lysine residues with glycine spacers (*p*[HPMA-*co*-(GK)_5_]) since increased charge densities were found to correlate with greater cytotoxicities [24]. Interestingly, these polymers induced similar transfection efficiencies than its pentamer counterpart (*p*[HPMA-*co*-K_5_]) but actually were more cytotoxic [25].

Arginine is another cationic amino acid residue that has been explored as a complexation agent. Arginine has been shown to exhibit cell-penetrating capabilities, thus allowing for enhanced cellular uptake [26]. Cell-penetrating peptides (CPPs), such as the TAT protein transduction domain, contain a number of arginine residues [27,28]. Harashima and coworkers demonstrated that liposomes modified with an increasing density of octaarginine (R_8_) peptides were taken up *via* macropinocytosis rather than clathrin-mediated endocytosis [29]. Therefore, arginine oligopeptides were also used as the complexation moiety in statistical HPMA brush polymers [25]. Similarly to previous reports comparing arginine motifs with lysine motifs [30-33], HPMA-oligoarginine polymers performed better in transfection efficiency when compared to a lysine analogue (~5-10 mmol lysine or arginine per gram polymer) but also caused increased cytotoxicity. Furthermore, due to challenges in synthesizing oligoarginine peptides, guanidinylation of the lysine resides with *O*-methylisourea was also explored; guanidinylation has been shown to increase transfection efficiencies of various carriers [34-37] and cellular uptake of HPMA constructs [38]. Conversion of the lysine residues in HPMA-oligolysine polymers to homoarginines resulted in higher transfection efficiencies and lower cytotoxicity than bPEI. Therefore, this method could be readily applied to other primary amine-based polymers to increase gene transfer efficiencies.

### Degradability using environmentally-responsive elements

Biodegradability is a desirable attribute for the *in vivo* application of gene delivery vectors. Since higher molecular weights of polycations can lead to increased cytotoxicity [23,24,39,40], linker chemistries have been used to introduce degradability into polymeric vectors. For example, environmentally-responsive linkages such as disulfide and acid-labile bonds can enable the release of cargo in specific intracellular compartments and promote degradability [41]. Likewise, specific amino acid sequences can be enzymatically degraded by various proteases [42-44]. We have explored both of these strategies for introducing a degradable segment into these HPMA-oligolysine polymers. Due to the relatively high levels of glutathione, a reducing agent, in the cytosolic environment compared to the extracellular space [45], the incorporation of disulfide linkages into polymeric carriers has been an attractive approach to increase biodegradability. To introduce reducibility, the six-carbon linker 6-aminohexanoic acid (Ahx) was exchanged with a linker containing a disulfide bond, 3-[(2-aminoethyl)dithio] propionic acid (Aedp) [46]. These reducible polymers were less cytotoxic, but achieved less efficient transfection efficiencies compared to the non-reducible analogue. However, a mixed formulation of reducible and non-reducible polymers achieved an intermediate level of transfection efficiency and reduced cytotoxicity. The high concentration of disulfide bonds within the polymer may lead to chemical instability, which was evidenced by partial improvement in transfection efficiency in the presence of EDTA.

As an alternative approach to enhancing degradability, we explored the use of enzymatically-cleavable peptide linkers, which have been used to introduce site-specific cleavage sites for the release of drugs and peptides [47-49]. A commonly used peptide linker sequence is cathepsin B-labile [12,50]; cathepsin B is a lysosomal cysteine protease that exhibits endo- and exopeptidase activity [51]. We designed a cathepsin B-labile peptide sequence (FKFL), and introduced the linker, flanked by six-carbon spacers (Ahx), between the HPMA backbone and the pendant cationic peptide [52]. The peptides demonstrated site-specific cleavage by cathepsin B within 15 minutes, while the polymers showed complete degradation of the pendant modified oligolysine motifs within 1 hour. In contrast to the reducible polymers in which transfection efficiencies were lower with polymers containing reducible linkers, the cathepsin B-labile polymers showed similar levels of transfection and were less toxic compared to a non-degradable analogue consisting of nondegradable d-amino acids. Therefore, this work demonstrates the possibility of using enzymatically-cleavable linkers to enable site-specific release and degradability for polyplex formulations.

### Endosomal escape strategies

Once internalized, polyplexes are exposed to increasingly acidic environments in endosomes and lysosomes, and eventually are degraded by lysosomal proteases. To circumvent lysosomal degradation, various strategies have been investigated to induce endosomal escape, such as the incorporation of peptide moieties that enable proton buffering [53] or interaction with lipid membranes [54]. Virally-derived peptides, such as TAT, Antennapedia (Antp), and HGP, and membrane-disruptive peptides, such as melittin, have been used to increase delivery efficiencies of cargo due to their ability to interact with lipid membranes [3]. We have used both of these approaches to enhance the endosomal escape abilities of our HPMA-oligolysine brush polymers, with varying success [55-57].

**Figure 3:**
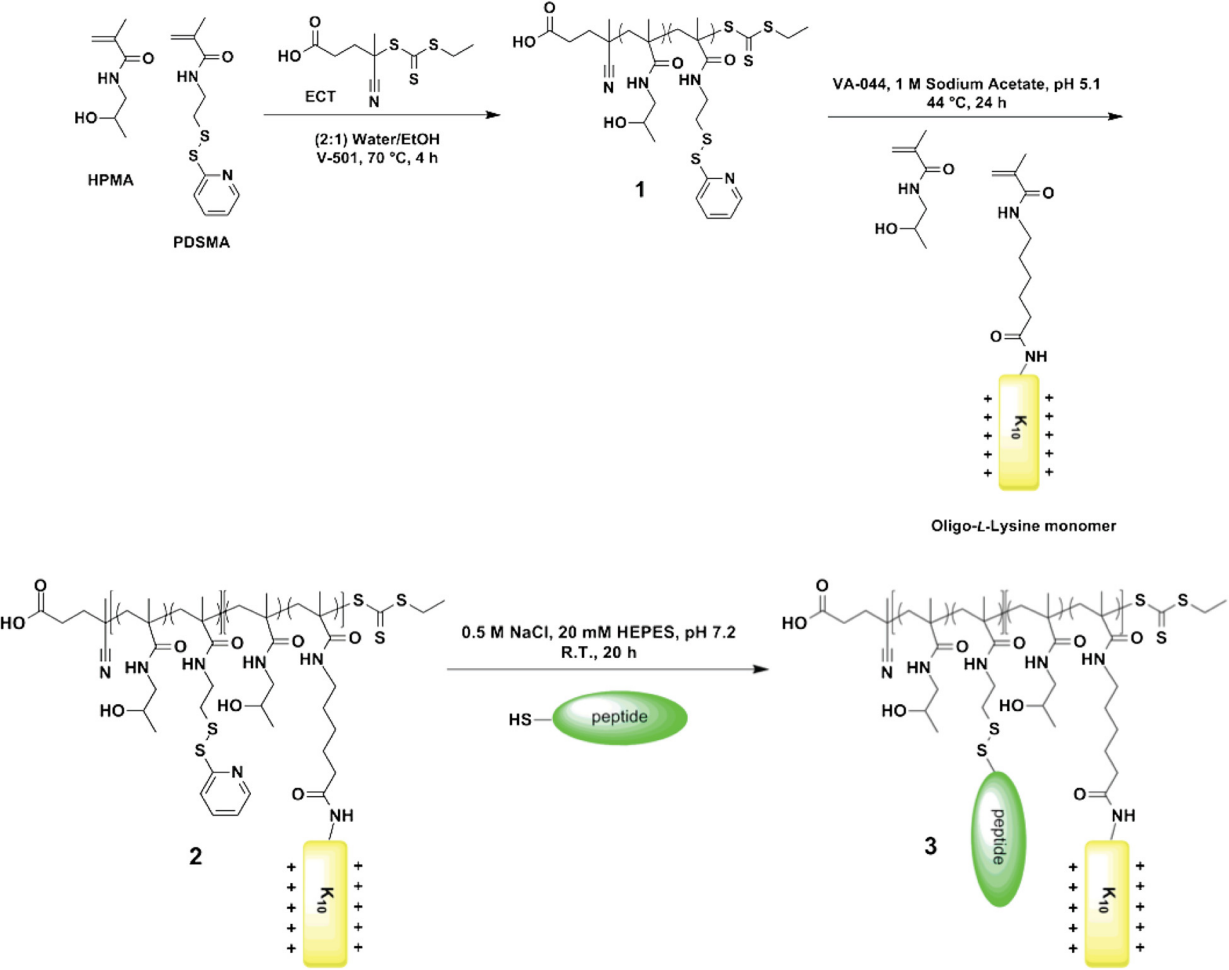
**Synthesis of diblock HPMA copolymers containing multiple pendant peptides.** RAFT polymerization of HPMA and PDSMA (molecule **1**), and then chain-extension with HPMA and oligo(l-lysine) to form the second block (molecule **2**). Disulfide exchange between the pyridyl disulfide on **2** and cysteine-functionalized oligo(l-histidine) peptides yields a diblock polymer with two bioactive peptides (molecule **3**).

As a first approach, we used a similar endosomal escape strategy to bPEI, which is commonly used as a transfection agent due to its ability to induce endosomal escape *via* the buffering of protons at pH ~6-7 [58,59]. Several groups have mimicked this buffering strategy by incorporating histidine residues, which contain a protonable imidazole group at pH 6–7, into various gene carriers [53]. The addition of a second oligohistidine-containing peptide into statistical HPMA-oligolysine polymers demonstrated increased transfection efficiencies [22]. To further optimize the incorporation of oligohistidine peptides to increase transfection, statistical and diblock polymers were synthesized with varying amounts of oligohistidine residues incorporated into the polymer (Figure 3) [55]. Interestingly, the polymer architecture affected the buffering range of the polymer in that diblock polymers buffered in the upper endosomal pH range (pH 5.6-7.4) whereas statistical polymers buffered in the lower endosomal pH range (pH 5.1-6.6). Despite improved buffering capabilities, only the statistical polymer containing 1.4 mmol histidine per gram polymer showed slight improvements in transfection ability over its non-histidylated analogue, possibly due to preferentially trafficking of the polyplexes through the non-acidifying caveolae-mediated endocytic pathway [60]. Furthermore, endosomal buffering requires a critical concentration threshold to be a viable strategy for endosomal escape, and thus, the use of more potent strategies may be beneficial.

**Figure 4:**
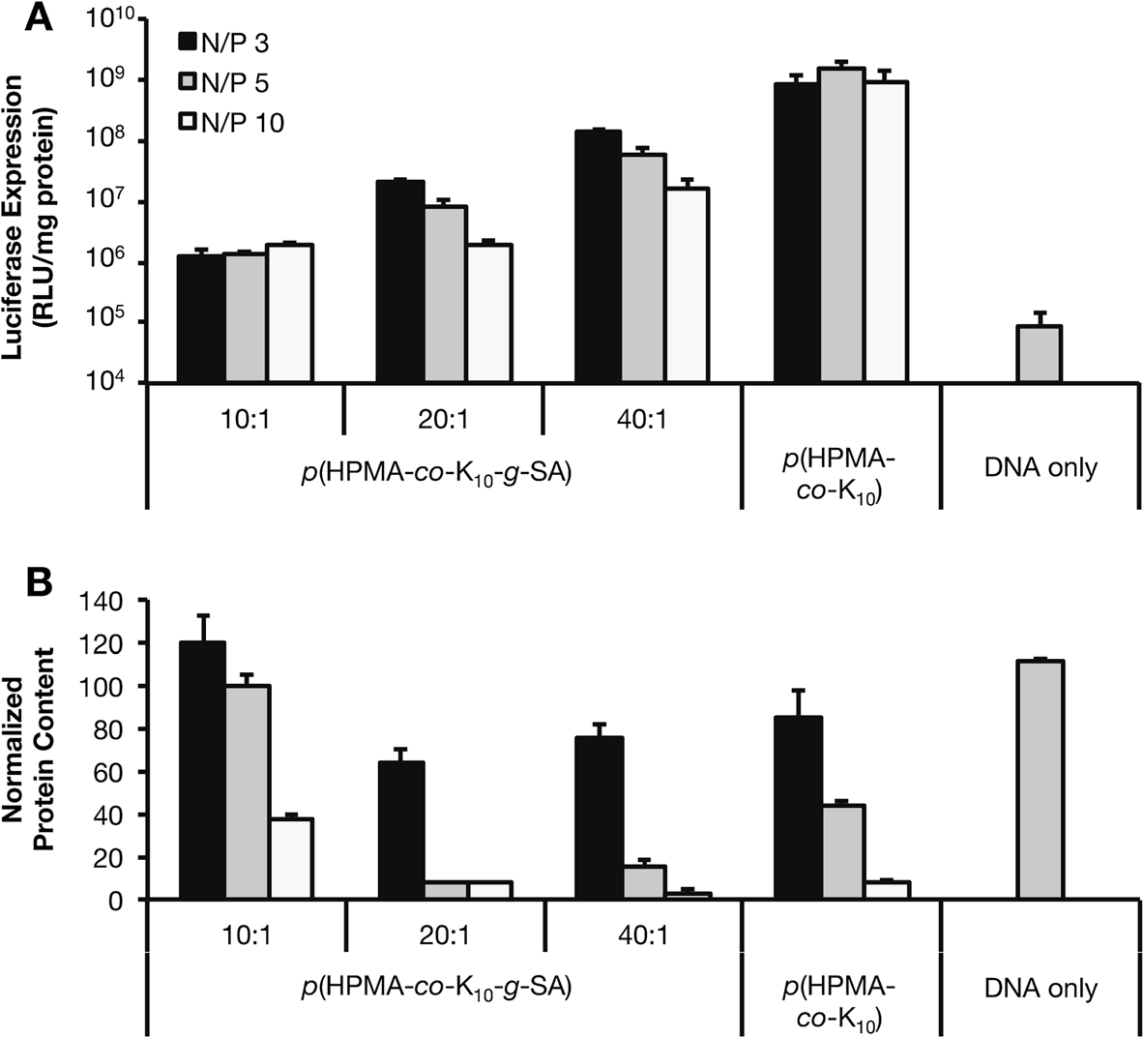
**Transfection of stearic acid (SA)-modified *p*[HPMA-*co*-K**_**10**_**] polyplexes in HeLa cells.** HeLa cells were treated with polyplexes (containing 1 μg plasmid DNA) at charge ratios (N/P) of 3, 5, and 10 for 4 h in serum-free conditions, washed, and replenished with complete media. At 48 h post-transfection, cell lysates were assessed for **(A)** luciferase reporter gene expression and **(B)** protein content as an indicator for cytotoxicity. Data are presented as the mean ± S.D., *n* = 3.

Hydrophobic modification of polycationic polymers is another strategy for promoting endosomal escape. Xu and Szoka proposed that upon endocytosis, lipid-modified polycation/DNA complexes form ion pairs with the negatively-charged amphiphilic endosomal membrane, resulting in membrane destabilization and release of cargo [61]. Furthermore, incorporation of hydrophobic moieties allows for physical encapsulation of the genetic payload, enhanced serum stability, increased cell viability, and targeting ability [62]. Several reviews have been published regarding the advantages of lipid or hydrophobic modification of polycationic gene carriers [63,64]. Previously, Abassi *et al.* reported various lipid-substituted polylysines as vectors for plasmid delivery and expression in skin fibroblasts. They showed that while all modified and unmodified polylysines demonstrated complete complexation with plasmid DNA, polymers with increased lipid substitution were more resistant to unpackaging with heparin treatment. Furthermore, most lipid-modified polymers exhibited increased pEGFP expression compared to native polylysine, with myristic and stearic acid lipid substituents demonstrating less cytotoxicity [65]. Inspired by this work, we prepared a panel of stearic acid-modified copolymers of HPMA and oligolysine. We grafted NHS-activated stearic acid (SA) onto the ϵ-amine of the lysine moiety of the previously optimized *p*(HPMA-*co*-K_10_) copolymers at DP 190. The lipid substituent was grafted at various lipid to lysine ratios for optimization (Table 1). We then evaluated the polymers for gene transfection. There was no significant increase in gene transfection with these lipid-modified copolymers *in vitro* (Figure 4A); increasing lipid substitution also decreased transfection efficiency (Figure 4B). Interestingly, high lipid substitution showed decreased cytotoxicity. Thus, for these HPMA-oligolysine polymers, lipid substitution is not a viable method for increasing transfection efficiency.

**Table 1:** **Characterization of lipid substitution on *p*** **[HPMA- *co *-K**_**10**_**] copolymers**

**Polymer designation**	**Lys:Lipid mole ratio**	Lipid/HPMA*	**% Substituted Lys**^**#**^
*p*(HPMA-*co*-K_10_-*g*-SA) (10:1)	10:1	0.35	14
*p*(HPMA-*co*-K_10_-*g*-SA) (20:1)	20:1	0.21	8.4
*p*(HPMA-*co*-K_10_-*g*-SA (40:1)	40:1	0.12	4.8

*Based on NMR integration of lipid –CH_2_-CO (~ 2.4 ppm) and HPMA –CH(OH)-CH_3_ (~ 3.7 ppm).

^*#*^Based on the theoretical mole ratio (~20% AhxK_10_ and 80% HPMA): 2.5 mole Lys/1 mole HPMA.

Alternatively, membrane-active peptides have been promising for inducing endosomal escape due to their high disruptive potency [54]. We have investigated the use of melittin, a 26-mer derived from venom of the honey bee *Apis mellifera*[56], and sHGP, a shortened and optimized 15-mer peptide derived from the endodomain of the HIV gp41 region [66]. Melittin undergoes an α-helical conformational change at lipid membranes, allowing the peptide to insert into the membrane and induce pore formation [67,68]. Polymeric carriers conjugated with melittin showed significantly increased transfection *in vitro* and *in vivo*[56,69,70]. Similarly to the HPMA-oligolysine-oligohistidine diblock copolymer, melittin was grafted onto a diblock of *p*[HPMA-*co*-PDSMA]-*b*-[HPMA-*co*-K_10_], using PDSMA as the point of conjugation [56]. Incorporation of melittin into these brush polymers displayed hemolytic ability and significantly improved transfection efficiency over the polymer without melittin incorporation, but also increased toxicity. To ameliorate some cytotoxicity, mixed formulations of the base polymer and the melittin-containing polymer were used for transfection and showed markedly improved toxicity profiles and even further improved transfection efficiencies to greater than that of branched PEI.

**Figure 5:**
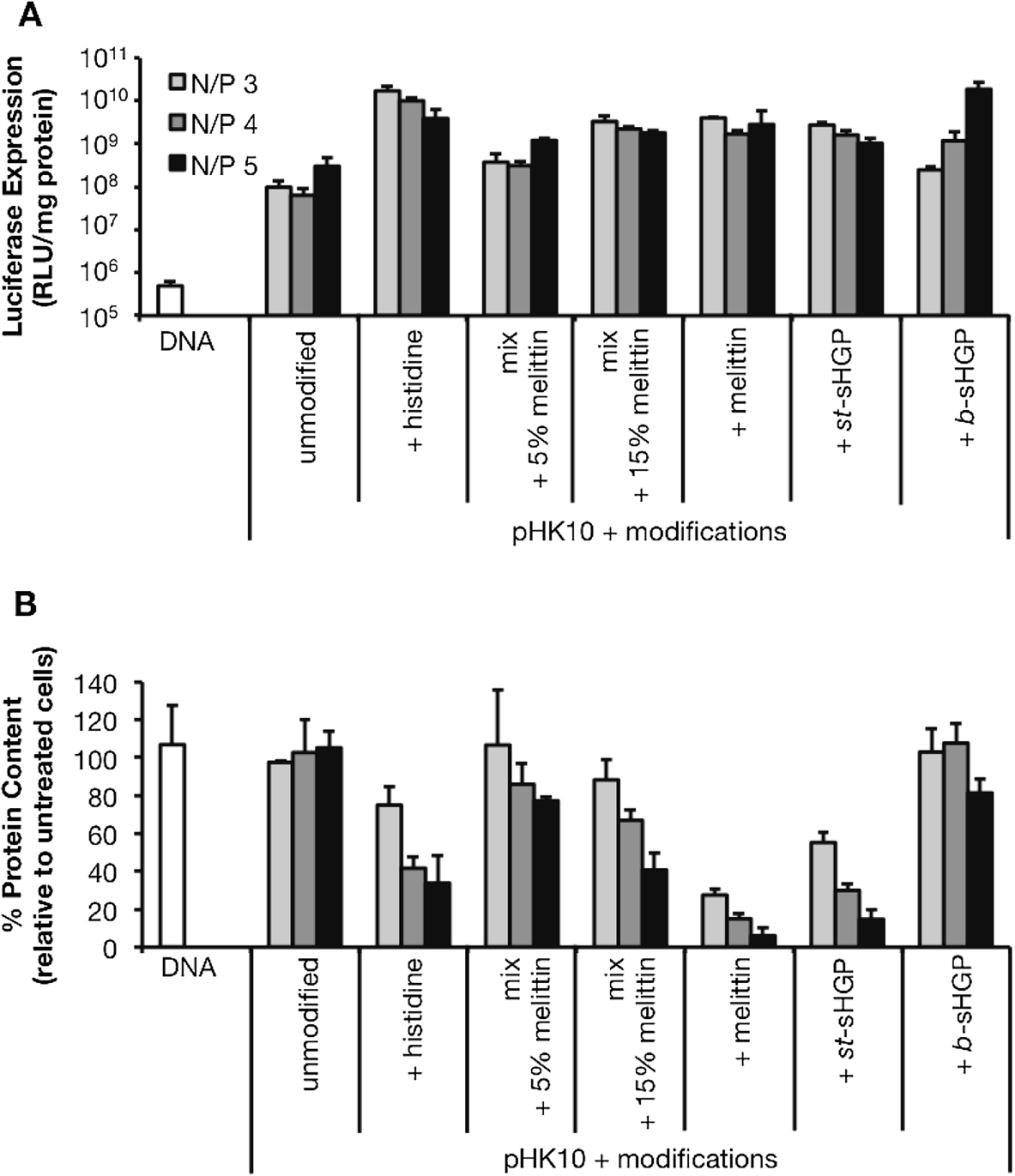
**Comparative transfection of *p*[HPMA-*co*-K**_**10**_**] polymers (“pHK10”) modified with various endosomal escape modalities in HeLa cells.** HeLa cells (3 × 10^4^) were treated with polyplexes (containing 1 μg plasmid DNA) at charge ratios (N/P) of 3, 4, and 5 for 4 h in serum-free conditions, washed, and replenished with complete media. At 48 h post-transfection, cell lysates were assessed for **(A)** luciferase reporter gene expression and **(B)** protein content as an indicator for cytotoxicity. *pHK10 unmodified*: *p*[HPMA-*co*-K_10_]; *pHK10 + histidine*: *p*[HPMA-*co*-K_10_-*co*-K_5_H_5_] [55]; *pHK10 mix + 5% melittin*: a mixture of 95:5% (v/v) *p*[HPMA-*co*-K_10_]: *p*[HPMA-*co*-melittin]-*b*-[HPMA-*co*-K_10_] [56]; *pHK10 mix + 15% melittin*: a mixture of 85:15% (v/v) *p*[HPMA-*co*-K_10_]: *p*[HPMA-*co*-melittin]-*b*-[HPMA-*co*-K_10_]; *pHK10 + melittin*: *p*[HPMA-*co*-melittin]-*b*-[HPMA-*co*-K_10_]; *pHK10 + st-sHGP*: *p*[HPMA-*co*-K_10_-*co*-sHGP]; *pHK10 + b-sHGP*: *p*[HPMA-*co*-sHGP]-*b*-[HPMA-*co*-K_10_]. Data are presented as the mean ± S.D., *n* = 3.

The incorporation of sHGP also helped increase the transfection efficiency of HPMA-oligolysine polymers [57]. sHGP was incorporated into HPMA-oligolysine polymers in a diblock architecture, similarly to melittin and the diblock oligohistidine-containing polymer, as well as a statistical architecture. Both polymer architectures demonstrated improved transfection efficiency compared to the base HPMA-oligolysine polymer, but the diblock polymer self-assembled into micelles, thereby sequestering the hydrophobic, membrane-lytic sHGP region. Interestingly, the diblock architecture resulted in less hemolytic activity and improved cytotoxicity profiles. Thus, these results suggest that the exposure of hemolytic regions under certain environmental conditions can lead to more efficient yet less toxic vectors. This strategy has previously been used in the design of synthetic polymers for siRNA delivery, where the exposure of a hydrophobic lytic domain occurred under endosomal pH to facilitate cargo release into the cytosol [71]. To compare these different endosomal escape strategies, cells were transfected with *p*[HPMA-*co*-K_10_] containing either oligohistidine, melittin, or sHGP peptides (Figure 5). All polymers that contained endosomal escape modalities performed better than the base polymer containing only oligolysine peptides. The statistical polymer containing oligohistidine peptides and the block polymer containing sHGP performed the best; however, the block sHGP polymer was the least toxic overall.

### Design considerations of HPMA-peptide polymers for gene delivery

Salt stability is necessary for the systemic administration of polyplexes since polyplex aggregation can lead to *in vivo* toxicity [72]. The incorporation of HPMA as a hydrophilic segment has been shown to reduce the salt-induced aggregation of multiple polymeric gene delivery systems [73-75]. We have also demonstrated increased salt stability of HPMA-oligolysine polyplexes when compared to lysine peptides or polylysine polyplexes [21-23]. In our optimization studies, salt stability decreased with increasing peptide incorporation [21,22], larger polymer molecular weights, and longer oligolysine peptide lengths [23]. In particular, the latter trend was unexpected since polymers containing longer oligolysine peptides also had longer HPMA segments in the polymer backbone. The incorporation of a hydrophilic HPMA shell onto *poly*(glycidyl methacrylate-tetraethylenepentamine) (*p*[GMA-TEPA]) also reduced salt-induced aggregation of the polyplexes; in this case, longer hydrophilic segments further enhanced polyplex stability in physiological salt conditions [76]. As seen with polymers containing poly(ethylene glycol) (PEG) to reduce polyplex aggregation under saline conditions [77], polymers containing the HPMA shell also demonstrated decreased *in vitro* transfection efficiencies. These results highlight the tradeoffs of incorporating hydrophilic segments into polymers for enhanced gene delivery *in vivo*.

In order to develop improved materials for nucleic acid delivery, it is important to understand the delivery mechanisms of engineered materials. To further characterize the HPMA-oligolysine polymers, we first determined the uptake efficiency of the optimized oligolysine-containing polymer (*p*[HPMA-*co*-K_10_]) (Shi J, Choi JL, Chou B, Johnson RN, Schellinger JG, Pun SH: Effect of polyplex morphology on cellular uptake, intracellular trafficking, and transgene expression. Submitted). Despite much lower polyplex uptake efficiencies of the optimized polymer (*p*[HPMA-*co*-K_10_]) *vs.* bPEI [78], the HPMA-oligolysine polymer achieved similar transfection efficiencies to that of bPEI [23]. Since alternative uptake routes have been implicated in improving polyplex transfection [60], transfection of the HPMA-oligolysine copolymers was also completed with the presence of various endocytic inhibitors [55]. Transfection efficiencies were decreased in the presence of a small-molecule inhibitor for caveolae-mediated endocytosis, a pathway that circumvents the acidification process necessary for endosomal buffering. These results suggest that transfection may be more productive when HPMA-oligolysine polyplexes are routed *via* a non-acidifying endocytic route, similarly to other polycation systems [60,79,80]. Therefore, understanding the uptake pathway of various polymer formulations can aid in the rationale design of improved materials.

### Therapeutic applications

#### Stealth coatings for adenovirus-mediated gene delivery

Adenoviruses, especially adenovirus serotype 5 (Ad5), are effective gene delivery agents since they are able to transduce both dividing and non-dividing cells, and can be produced in large titers [81,82]. However, Ad5 also induces a host immune response, limiting its use for systemic re-administration, and can only transduce cells expressing Coxsackievirus and Adenovirus Receptor (CAR) and αV integrins on their surface. To reduce the immunogenic response to the Ad5 capsid, as well as enhance the uptake of Ad5 in CAR-negative cell types, synthetic polymers using polyethylene glycol (PEG) and HPMA have been explored as stealth coatings [83]. We have also used HPMA-oligolysine brush polymers, with varying oligolysine peptide lengths, polymer molecular weights, and linker degradability properties, to electrostatically interact with the negatively-charged viral capsid to form stealth coatings [84]. A polymer with a peptide length of ten lysines was optimal for increasing transduction of Ad5 under serum and serum-free conditions; interestingly, a similar polymer was also found to be optimal for nonviral polyplex transfections [23]. The polymer coatings also enabled transduction of CAR-negative cells, which was mediated by the presence of heparan sulfate proteoglycans (HSPGs) on the cell surface. In particular, sulfated HSPGs have been implicated in enhancing cellular uptake of cationic materials through electrostatic interactions [85]. Furthermore, the stealth coating also significantly protected the virus from neutralizing antibodies, which inhibits efficient *in vivo* viral transduction, without reducing transduction efficiency; these results demonstrated the potential applicability of HPMA-oligolysine brush polymers to enhance Ad5 transduction *in vivo*.

#### Delivery to the central nervous system (CNS)

The delivery of therapeutic genes to the CNS can be beneficial for the treatment of neurological disorders [86,87]. However, gene delivery to the CNS using nonviral vectors has not been successful due to the low transfection efficiencies achieved in non-dividing cells and low cellular uptake rates in terminally-differentiated neurons [88-90]. One approach to increase efficacy is to conjugate targeting ligands to polymeric vectors to enhance uptake by neuronal subtypes [7,91,92]. We previously demonstrated that conjugation of Tet1, a 12-mer peptide identified by *in vitro* phage display to bind the *Gtb1* ganglioside [93], to polyethylenimine (PEI) enhanced gene delivery to neural progenitor cells (NPCs) [94]. We found that ~0.6 Tet1 per polymer was sufficient to enhance transfection efficiency to NPCs *in vivo*. Recently, we have extended this work by exploring the effect of multivalency on targeting capability by varying ligand density of pendant Tet1 peptides in statistical HPMA-oligolysine copolymers [95]. We showed that there was an optimal ligand density for transfection of neuron-like PC-12 cells (~3 Tet1 per polymer), mainly due to increased toxicities seen with higher Tet1 ligand incorporation. Interestingly, high concentrations (> 100 μM) of the Tet1 peptide alone did not cause significant cytotoxicity, suggesting that the incorporation of hydrophobic peptides such as Tet1 can increase the toxicity of cationic polymers [63].

Another approach for increasing transfection efficiency to neurons is to enhance endosomal escape of endocytosed polyplexes [96]. We have also demonstrated that melittin-containing HPMA polymer carriers enhanced effective bulk luciferase transfection to the brain over polymers without melittin after intraventricular injection [56].

### Conclusions and future directions

In summary, we have demonstrated the synthesis of cationic polymers consisting of a hydrophilic HPMA backbone with multiple pendant peptides for DNA condensation, degradability, and endosomal escape. These HPMA-peptide brush polymers have also been used to improve adenovirus transduction and targeted delivery to the central nervous system. The relatively simple polymerization strategy used to synthesize these polymers has allowed the investigation of peptide multivalency, polymer architecture, and the use of various bioactive peptides for gene delivery applications. Currently, we are applying our findings from these described studies to improve other gene delivery systems for *in vivo* delivery. More broadly, our enhanced understanding of the delivery mechanisms of these HPMA-peptide materials is applicable to other gene delivery systems. Furthermore, these versatile materials have shown potential for the general delivery of biologics, such as therapeutic peptides and proteins.

## Competing interests

The authors declare that they have no competing interests.

## Authors’ contributions

JS compared the transfection efficiencies of polymers modified with endosomal release moieties, JGS synthesized and tested lipid-modified carriers, and JS and SHP prepared the manuscript. All authors read and approved the final manuscripts.
